# Crimean-Congo Hemorrhagic Fever Virus for Clinicians—Virology, Pathogenesis, and Pathology

**DOI:** 10.3201/eid3005.231646

**Published:** 2024-05

**Authors:** Maria G. Frank, Gretchen Weaver, Vanessa Raabe, State of the Clinical Science Working Group of the National Emerging Pathogens Training and Education Center’s Special Pathogens Research Network

**Affiliations:** Denver Health and Hospital Authority, Denver, Colorado, USA (M.G. Frank);; University of Colorado School of Medicine, Denver (M.G. Frank);; University of Massachusetts Chan Medical School, Worchester, Massachusetts, USA (G. Weaver);; New York University Grossman School of Medicine, New York, New York, USA (V. Raabe)

**Keywords:** Crimean-Congo hemorrhagic fever, viruses, vector-borne infections, bunyavirus, viral hemorrhagic fever, countermeasure, vaccine, treatment

## Abstract

Crimean-Congo hemorrhagic fever (CCHF), caused by CCHF virus, is a tickborne disease that can cause a range of illness outcomes, from asymptomatic infection to fatal viral hemorrhagic fever; the disease has been described in >30 countries. We conducted a literature review to provide an overview of the virology, pathogenesis, and pathology of CCHF for clinicians. The virus life cycle and molecular interactions are complex and not fully described. Although pathogenesis and immunobiology are not yet fully understood, it is clear that multiple processes contribute to viral entry, replication, and pathological damage. Limited autopsy reports describe multiorgan involvement with extravasation and hemorrhages. Advanced understanding of CCHF virus pathogenesis and immunology will improve patient care and accelerate the development of medical countermeasures for CCHF.

Crimean-Congo hemorrhagic fever (CCHF) was clinically categorized as a disease during World War II when, after being exposed to ticks, ≈200 soldiers from the Soviet Union stationed in the Crimean Peninsula during 1944–1945 developed hemorrhagic fever symptoms, as part of an illness initially termed Crimean hemorrhagic fever. Similar clinical features had been described in present-day Tajikistan and Uzbekistan as early as the 12th Century (*1*,*2*). An enveloped, single-stranded RNA virus isolated from an infected patient in 1967 was named Crimean hemorrhagic fever virus. Another virus (Congo virus) was identified following a hemorrhagic fever outbreak in the current Democratic Republic of the Congo (formerly Zaire) in 1956 (*2*). In the early 1970s, the name was changed to Crimean-Congo hemorrhagic fever virus (CCHFV), after Crimean hemorrhagic fever virus and Congo viruses were found to be serologically indistinguishable in 1967. 

Human CCHFV infection mainly occurs through the bite of an infected tick or exposure to blood or tissue from infected animals; human-to-human transmission, particularly in healthcare settings, has been reported (*3*–*5*). Approximately 10,000–15,000 CCHF cases are estimated to occur worldwide each year, but more definitive numbers are difficult to ascertain. Uncertainty arises because up to 88% of cases are thought to be subclinical (*6*–*8*), unrecognized, or occur in locations with limited disease surveillance or laboratory testing capability; also, the case definition for CCHF is not standardized across endemic regions (*9*,*10*). A recent worldwide systematic review and meta-analysis, using data collected during 1974–2020, reported an overall case-fatality rate of 11.7% for humans with acute CCHFV infection (defined as presence of live virus, viral antigen, or RNA), a prevalence of 22.5% (n = 35,198), recent infection (defined as presence of IgM) seroprevalence of 11.6% (n = 27,173), and an overall past infection (defined as presence of IgG) seroprevalence of 4.3% (n = 74,900) in humans (*11*). 

CCHFV is an enveloped, multisegmented, single-stranded, negative-sense RNA virus (genus *Orthonairovirus*, order Bunyavirales, family Nairoviridae). The viral genome exists as 3 single-stranded, negative-sense RNA molecules, leading to a complex replication program. Replication of the trisegmented CCHFV genome is error prone, leading to antigenic drift resulting in 7 distinct genotypes (*12*). CCHFV binds to an unknown cell receptor; however, the low-density lipoprotein receptor (LDLR) has recently been proposed as critical for CCHFV cell entry (*13*). The virus can enter a wide range of human cells, triggering damage both directly as a result of viral infection and indirectly by modifying vascular permeability and eliciting a proinflammatory immune response (*14*). 

Disease because of CCHFV infection is limited to humans, although asymptomatic transient viremia lasting up to 15 days has been documented in multiple livestock and wild animals (*15*). Severe or fatal human disease correlates with an exuberant proinflammatory immune response leading to vascular dysfunction, disseminated intravascular coagulation, multiorgan failure, and shock (*16*). Detection of IgM, usually present as early as 4–5 days after illness onset, and IgG, usually present 7–9 days after illness onset, correlate with declining viremia (*17*). However, antibody response to CCHFV does not correlate with disease outcomes or protection through vaccination (*17*). 

This first article in a 3-part series summarizing the main aspects of CCHF is meant to provide clinicians with an overview of the virology, pathogenesis, and pathology of CCHF. The second article focuses on epidemiology, clinical features, and prevention and control of CCHF (*18*) and the third on diagnostic testing and management of CCHF (*19*). 

## Methods

The focused review for this paper involved MeSH (National Center for Biotechnology, https://www.ncbi.nlm.nih.gov/mesh) and PubMed (https://pubmed.ncbi.nlm.nih.gov) search strings customized for CCHF/CCHFV. We focused our review on human data from the past 10 years when available; we included older data or data from animal cases where appropriate. We conducted title, abstract, and full text reviews of relevant manuscripts, reviews, and book chapters. We also completed bibliography scans on reviewed articles and meta-analyses. 

### Virology

CCHFV virions are pleiomorphic, but mostly spherical, and measure 80–120 nm in diameter (*2*). The natural cycle of CCHFV involves both domestic and wild animals as hosts, with ticks from *Hyalomma* (CCHFV’s main vector), *Rhipicephalus*, and *Dermacentor* genera as vectors and reservoirs (*12*). The natural cycle includes transovarial (vertical) and transstadial (horizontal) transmission among ticks and transmission between ticks and their vertebrate hosts. Humans are considered dead-end or accidental hosts for the virus because they are not a source of infection for ticks. CCHFV virions contain a trisegmented, negative-sense RNA genome comprised of the small (S) segment, which encodes the nucleocapsid protein (NP) and nonstructural protein (NS); medium (M) segment, which encodes membrane glycoproteins Gn and Gc as well as several NS; and large (L) segment, which encodes the RNA-dependent RNA polymerase (RdRp) (*7*,*20*,*21*). 

The S segment (NSs) encodes the NP, which is composed of a globular domain and protruding arm, and a small NS (*12*). The NP interacts with viral RNA to form ribonucleoprotein (RNP) complexes. The NP also performs endonuclease activity that promotes viral replication, transcription, and assembly, and interacts with host heat shock proteins during intracellular replication of the virus (*22*,*23*). It has been postulated that both NP and NS might have a role in cellular apoptosis as well (*22*).

The M segment encodes a polyprotein that results in 2 transmembrane glycoproteins, Gn and Gc, and NS, such as GP160/85 that is further processed into GP38, a mucin-like domain (MLD), and M-segment nonstructural protein (NSm) after cleavage (*12*,*21*,*22*). Gn and Gc stud the virion lipid envelope as spikes. Gc is assumed to be responsible for binding to cellular receptors and has recently been described binding to the LDLR present in various human cells; of note, the LDLR density is directly correlated with CCHFV infectivity (*13*). Gc has been identified as the target for neutralizing antibodies generated during the infection course as well (*17*). Gn contributes to membrane fusion (*17*). MLD and GP38 may play roles in glycoprotein processing and incorporation into virions (*23*). Both NSs and NSm have been postulated to have roles in interferon antagonism (*17*,*24*).

The L segment encodes a single, large protein containing RdRp enzyme and cap-snatching mechanisms required for genome replication (*23*,*26*). The RdRP protein also harbors an ovarian tumor protease (OTU) that may function as an inhibitor of the multiple host-cell antiviral mechanisms in the interferon-signaling pathway (*12*,*27*,*28*). 

As for most viruses in the Nairoviridae family, the replication cycle for CCHFV commences after the binding of the viral glycoprotein, Gc for CCHFV, to a host cell receptor, potentially LDLR, leading to receptor-mediated endocytosis (*13*). Reduced pH in the endosome provokes a change in glycoprotein morphology with consequent fusion of endosomal membrane and envelope resulting in release of ribonucleoprotein into cytosol. The genomic ribonucleoprotein acts as the template for RdRp-generating virus mRNA, which is translated into viral proteins and cRNA which serves as the template for genomic viral RNA (vRNA) production. New ribonucleoprotein is formed by association of vRNA, RdRp, and capsid proteins; glycoprotein translation and cleavage into Gc and Gn precursors occur in the endoplasmic reticulum. Further processing and maturation of glycoproteins happens in Golgi complex completing assembly of new virions. Once virion assembly and transport to plasma membrane is complete, virions are released through exocytosis (*1*,*12*,*29**,**30*). 

CCHFV is a genetically diverse virus with 20% sequence divergence of S segment, 31% of M segment, and 22% of L segment of virus isolates (*12*). Based on S segment sequence data, 7 CCHFV genotypes correlate with the geographic area of parent virus identification, hence the terminology used by Atkinson to name the different genotypes: Africa 1–3, Asia 1 and 2, and Europe 1 and 2 (*29*,*31*). Those lineages correlate with Carroll’s denomination into 6 clades: I (Africa 3), II (Africa 2), III (Africa 1), IV (Asia 1 and 2), V (Europe 1), and VI (Europe 2) (*32**,**33*). Research has shown that CCHFV evolves and acquires genetic diversity through various mechanisms. The virus can accumulate mutations through antigenic drift from a common ancestor. Further, the multisegmented genome enables reassortment events when coinfection with 2 different strains occurs, resulting in a dramatic antigenic shift. Reassortment is especially concerning because it can increase through expanded travel and long-range transport of infected ticks or animals. Finally, there is evidence of recombination between RNA segments of different strains (*12*). Although there is greater genetic diversity within M segment than L and S segments, resulting in Gn and Gc nucleotide diversity, this difference does not render a greater antigenic variety (*12*,*33*). 

It has been proposed that differences between viral lineages and their adaptation to regional hosts might affect the severity of human illness (*12*). The AP92 strain of CCHFV was recovered from a *Rhipicephalu*s sp. tick in Greece in 1979 (*34*). Based on indirect epidemiologic data, AP92 is thought to be avirulent or have very low virulence in humans. Similar strains have recently been isolated in Turkey and other areas from patients with mild CCHF (*34**–**36*). In contrast, patients in South Africa infected with a reassorted CCHFV strain (M segment mapped to Asian clades, S and L segments mapped to African clades) suffered a higher mortality rate when compared with patients infected with the nonreassorted endemic CCHFV strains (*37*). 

## Pathogenesis 

Although CCHF pathogenesis and immunobiology are not yet fully understood, multiple processes seem to contribute to viral entry, replication, and immune response (Figure). After transmission from an infected tick, CCHFV passes through the epithelium into the basolateral compartment of the skin, where it infects endothelial cells of local capillaries and small blood vessels, dendritic cells, and macrophages (*38*). Viral entry is mediated by the Gc component of the envelope protein, which binds to a host cell receptor, likely LDLR, for entry (*13*,*17*).

**Figure:**
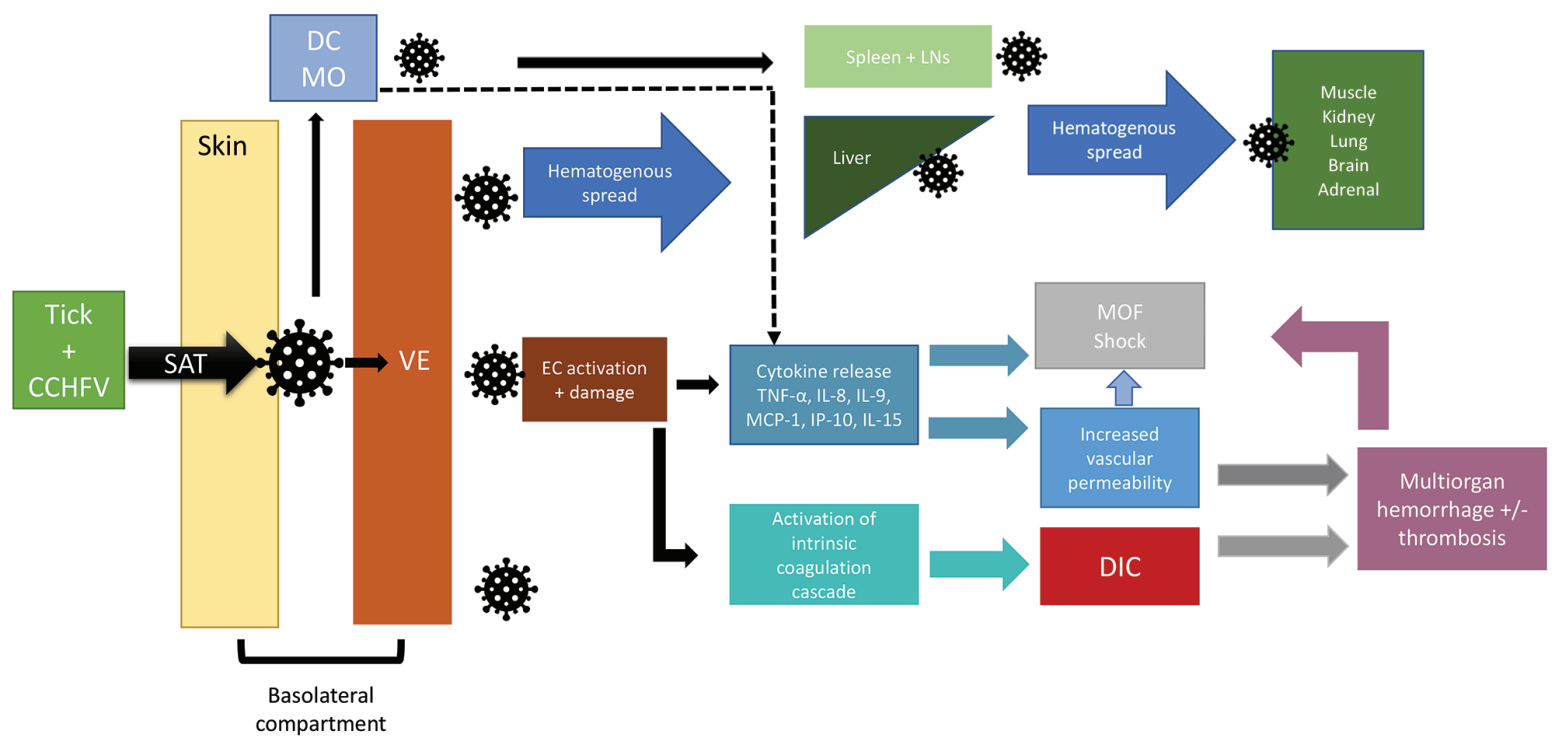
Flowchart showing an abbreviated proposed pathway for Crimean-Congo hemorrhagic fever virus pathogenesis. CCHFV, Crimean-Congo hemorrhagic fever virus; DC, dendritic cell; DIC, disseminated intravascular coagulation; EC, endothelial cell; IL, interleukin; LN, lymph nodes; MCP, monocyte chemoattractant protein; MO, macrophage; MOF, multiorgan failure; SAT, saliva-assisted transmission; TNF, tumor necrosis factor; VE, vascular endothelium.

The shorter CCHF incubation period associated with tick-mediated exposure, as compared with exposure to blood and tissue of infected animals, has been attributed to tick saliva–assisted transmission (SAT) mediating viral entry and replication. Tick saliva contains a mixture of peptide and nonpeptide molecules, as well as water, ions, host proteins, and exosomes (*39*,*40*). Multiple tick saliva components may contribute to SAT by counteracting host-derived vasoconstrictors, inhibiting multiple host cell responses including wound healing, complement pathways, platelet aggregation, local coagulation pathways, and promoting local analgesia by means of bradykinin inhibition (*40**–**42*). The specific tick saliva components mediating SAT might vary depending on the specific tick species. 

In vitro, CCHFV replicates in human cell lines from adrenal, bone marrow, brain, cervix, liver, lung, lymphocytes, kidney, muscle, and vascular endothelium (*35*). In vivo, after initial entry and replication, CCHFV spreads hematogenously, leading to potential infection in multiple organs: adrenals, liver, lungs, spleen, and kidneys (*14*,*44*,*45*). Infection of glial cells and astrocytes has been documented in humanized mouse models, but not to date in human patients (*46*). 

CCHFV infection results in both direct cellular damage, such as apoptosis, and indirect damage, such as increased vascular permeability through upregulation of soluble adhesion molecules (i.e., E-selectin, vascular cell adhesion molecule 1 [VCAM 1], intracellular adhesion molecule 1 [ICAM 1], and vasoactive molecules) (*14*,*43*). Vascular endothelial damage promotes platelet aggregation and degranulation leading to subsequent activation of the intrinsic coagulation cascade which, in severe cases, culminates in disseminated intravascular coagulation (*14*). CCHFV infection also leads to the release of proinflammatory cytokines which can lead to immune-mediated damage. Robust proinflammatory response has been associated with severe cases and fatal outcomes (*17*). Specifically, positive associations between disease severity and poor prognosis have been correlated with elevated levels of interleukin (IL) 8, IL-9, IL-15, IP-10, TNF-α (tumor necrosis factor-α), and MCP 1 (monocyte chemoattractant protein 1) (*17*). In contrast, RANTES (regulated upon activation, normal T cell expressed and secreted; also called CCL-5 [chemokine ligand 5]) levels appear to have a negative correlation with severity of CCHF (*17*). CCHFV is an interferon-susceptible virus; infected cells delay induction of type-1 interferon, giving the virus time to replicate and spread systemically (*47*). 

Immune correlates of protection against and resolution of CCHF remain unknown. IgM and IgG responses are associated with declining viremia, and the generation of an antibody response is associated with better disease outcomes. However, the role of antibodies in controlling infection remains unclear (*17*). It is well documented that severe CCHF cases have minimal humoral immune response (*12*,*48*). Conversely, survivors develop CCHFV-specific humoral and cellular immunity, and to date, reported human reinfection has not been documented (*17*,*27*,*49*,*50*). Studies from CCHFV-infected nonhuman primates suggest that antibody titers and neutralizing activity do not correlate well with severity of disease or outcomes (*45*). 

## Pathology

To date, few autopsy or necropsy reports of CCHF patients have been published. Histopathologic reports on 2 skin biopsies noted diffuse extravasation of erythrocytes into the epithelial interstitium, associated with hemorrhages in the skin (Appendix reference *51*). Anecdotally, a liver biopsy obtained during a nosocomial outbreak in South Africa in 1984 showed interhepatocyte infiltration of erythrocytes with diffuse extravasation (Appendix reference *51*). Using electron microscopy, evidence of pericapillary edema and autolysis of hepatocytes, as well as intracytoplasmatic virions in epithelial cells of hepatic sinusoids and portal vessels, were observed (Appendix reference *51*). Evidence of hepatic lesions ranged from disseminated necrosis to multiple necrotic foci. Of note, the foci of viral antigen demonstrated by immunofluorescence was disproportionate to the severity of necrosis, suggesting injury mechanisms other than direct viral cytopathic effect. Thrombus formation in central and portal veins was found in patients with more severe liver involvement (Appendix reference *52*). More recent reviews of liver pathology using immunohistochemistry demonstrated infection of Kupffer cells, hepatic endothelial cells, and hepatocytes, with mononuclear portal inflammation, and necrosis characterized by hemorrhage (*14*). A marked splenic lymphoid apoptosis and lymphocyte depletion with dilated sinusoids were also described (*14*).

Petechial hemorrhage of serosa, liver and spleen capsule, and intestinal hyperemia are visible by macroscopic exam (*12*). Necropsy from an autochthonous case in Spain, in addition to hepatocyte necrosis, demonstrated cytoplasmic macrovesiculation and microvesiculation, complete epithelial denudation of the colon, occasional microthrombi, and bone marrow hemorrhages (Appendix reference *53*). A study from Turkey of 5 confirmed and 14 suspected CCHF cases demonstrated hemophagocytosis with unclear clinical significance in the bone marrow of 7 patients (Appendix reference *54*). 

## Conclusions

CCHFV is an enveloped, single-stranded RNA, tickborne virus. The virus life cycle and molecular interactions are complex and not fully described. Although pathogenesis and immunobiology are not yet fully understood and research is needed to fill the gaps, it is clear multiple processes contribute to viral entry, replication, and pathological damage, and limited autopsy reports describe multiorgan involvement with extravasation and hemorrhages. Broadening knowledge about CCHFV pathogenesis and immunology will enable improved patient care and accelerate the development of medical countermeasures for CCHF. 

Appendix Additional references from summary of Crimean-Congo hemorrhagic fever virus for clinicians.
