# Supplementary material for: Crimean-Congo Hemorrhagic Fever Virus for Clinicians—Virology, Pathogenesis, and Pathology
Source: Emerg Infect Dis. 2024 May;30(5):847–53. doi: 10.3201/eid3005.231646 (PMC11060449; doi:10.3201/eid3005.231646)
Supplement: Appendix — Additional references from summary of Crimean-Congo hemorrhagic fever virus for clinicians. [file 23-1646-Techapp-s1.pdf]

Article DOI: https://doi.org/10.3201/eid3005.231646

Crimean Congo Hemorrhagic Fever Virus for Clinicians—Virology, Pathogenesis, and Pathology

Additional References

51. Joubert JR, King JB, Rossouw DJ, Cooper R. A nosocomial outbreak of Crimean-Congo haemorrhagic fever at Tygerberg Hospital. Part III. Clinical pathology and pathogenesis. S Afr Med J. 1985;68:722–8. PubMed

52. Swanepoel R, Gill DE, Shepherd AJ, Leman PA, Mynhardt JH, Harvey S. The clinical pathology of Crimean-Congo hemorrhagic fever. Rev Infect Dis. 1989;11(Suppl 4):S794–800. PubMed https://doi.org/10.1093/clinids/11.Supplement_4.S794

53. Negredo A, de la Calle-Prieto F, Palencia-Herrejón E, Mora-Rillo M, Astray-Mochales J, Sánchez-Seco MP, et al.; Crimean Congo Hemorrhagic Fever@Madrid Working Group. Autochthonous Crimean-Congo hemorrhagic fever in Spain. N Engl J Med. 2017;377:154–61. PubMed https://doi.org/10.1056/NEJMoa1615162

54. Karti SS, Odabasi Z, Korten V, Yilmaz M, Sonmez M, Caylan R, et al. Crimean-Congo hemorrhagic fever in Turkey. Emerg Infect Dis. 2004;10:1379–84. PubMed https://doi.org/10.3201/eid1008.030928
